# Vascular endothelial growth factor isoforms differentially protect neurons against neurotoxic events associated with Alzheimer’s disease

**DOI:** 10.3389/fnmol.2023.1181626

**Published:** 2023-06-27

**Authors:** Roaa H. Alalwany, Tom Hawtrey, Kevin Morgan, Jonathan C. Morris, Lucy F. Donaldson, David O. Bates

**Affiliations:** ^1^Tumour and Vascular Biology Laboratories, Division of Cancer and Stem Cells, Centre for Cancer Sciences, School of Medicine, Biodiscovery Institute, University of Nottingham, Nottingham, United Kingdom; ^2^School of Chemistry, University of New South Wales, Sydney, NSW, Australia; ^3^School of Life Sciences, University of Nottingham, Nottingham, United Kingdom; ^4^Pan African Cancer Research Institute, University of Pretoria, Pretoria, South Africa

**Keywords:** Alzheimer’s disease, VEGF, splicing, tau, SRPK1, amyloid-beta

## Abstract

Alzheimer’s disease (AD) is the most common cause of dementia, the chronic and progressive deterioration of memory and cognitive abilities. AD can be pathologically characterised by neuritic plaques and neurofibrillary tangles, formed by the aberrant aggregation of β-amyloid and tau proteins, respectively. We tested the hypothesis that VEGF isoforms VEGF-A_165_a and VEGF-A_165_b, produced by differential splice site selection in exon 8, could differentially protect neurons from neurotoxicities induced by β-amyloid and tau proteins, and that controlling expression of splicing factor kinase activity could have protective effects on AD-related neurotoxicity *in vitro*. Using oxidative stress, β-amyloid, and tau hyperphosphorylation models, we investigated the effect of VEGF-A splicing isoforms, previously established to be neurotrophic agents, as well as small molecule kinase inhibitors, which selectively inhibit SRPK1, the major regulator of VEGF splicing. While both VEGF-A_165_a and VEGF-A_165_b isoforms were protective against AD-related neurotoxicity, measured by increased metabolic activity and neurite outgrowth, VEGF-A_165_a was able to enhance neurite outgrowth but VEGF-A_165_b did not. In contrast, VEGF-A_165_b was more effective than VEGF-A_165_a in preventing neurite “dieback” in a tau hyperphosphorylation model. SRPK1 inhibition was found to significantly protect against neurite “dieback” through shifting AS of *VEGFA* towards the VEGF-A_165_b isoform. These results indicate that controlling the activities of the two different isoforms could have therapeutic potential in Alzheimer’s disease, but their effect may depend on the predominant mechanism of the neurotoxicity—tau or β-amyloid.

## 1. Introduction

The neurovascular unit (NVU) controls blood–brain barrier (BBB) permeability and cerebral blood flow, maintaining the chemical composition of cerebral tissue required for its proper function (Zlokovic, 2011). The NVU unit comprises vascular cells (endothelium, pericytes and smooth muscle), glial cells and neurones. Dysfunction of the NVU, and resulting disruption to the BBB, is associated with the accumulation of neurotoxins, including β-amyloid, as well as a reduction in cerebral blood flow and hypoxia. Cerebrovascular deficits are common in AD: >50% of patients have ischaemic white matter damage and > 90% of patients have cerebral amyloid angiopathy, characterised by the deposition of β-amyloid in vessel walls (Thomas et al., 2015). Furthermore, vascular risk factors, such as atherosclerosis and diabetes mellitus, also increase risk of developing AD (Yang et al., 2004).

There is growing evidence that amyloid production and cerebral hypoperfusion are inherently linked to each other in AD pathology. VEGF-A, a key regulator of angiogenesis, is known to bind to amyloid peptides with high affinity (50 pM; Yang et al., 2004) and specificity. In addition, it has been found to co-localise with amyloid plaques in the AD brain (Yang et al., 2004). β-amyloid treatment has been shown to alter angiogenesis in a concentration dependent manner: At lower concentrations, amyloid can result in the stimulation of microvessel formation whereas higher concentrations significantly inhibit the angiogenic process in endothelial cells (Paris et al., 2004). Investigation of amyloid’s anti-angiogenic activity found that it can inhibit VEGFR2 activation by directly binding and acting as an antagonist of the receptor (Patel et al., 2010). Furthermore, β-amyloid treatment of endothelial cells can reverse a VEGF-A induced increase in permeability. Considering VEGF-A is a very potent permeability factor, these studies put forward evidence of amyloid’s ability to antagonise VEGF-A related activity.

Interestingly, levels of VEGF-A are increased in the AD brain, specifically in the frontal cortex and para-hippocampus (Thomas et al., 2015). In addition, the total level of VEGFR1, a negative regulator of VEGF-A expression, is reduced in AD despite being upregulated by hypoxia (Harris et al., 2018). However, this may be offset by the fact the ratio of membrane bound to soluble VEGFR1 is also reduced, since soluble VEGFR1 is thought to be signalling inactive and can sequester VEGF-A by acting as a competitive inhibitor of its ligand. Ultimately, this suggests that inhibition of VEGF-A binding at VEGFR2, and related VEGF-A mediated activity, may be driving upregulation of its expression. Indeed, Thomas et al. (2015) suggested that overproduction of VEGF-A in AD may be a compensatory mechanism for reduced cerebral perfusion associated with the accumulation of amyloid. This is supported by evidence that VEGF-A expression positively correlates with disease severity (Thomas et al., 2015). Since cerebral hypoperfusion persists as a symptom of AD, endogenous upregulation of VEGF-A may be an insufficient response to NVU dysfunction. Exogenous VEGF-A can oppose the effect of high amyloid concentrations and partially restore angiogenic activity *in vitro* (Patel et al., 2010) suggesting that stimulation of VEGF-A activity could be relevant to neuroprotection. However, it is important to elucidate the direct effect on neurones as the majority of studies so far have only characterised role of VEGF-A in the endothelial cell component of the NVU.

As a hallmark of AD pathology, neurofibrillary tangles (NFTs) are the second key driver of cognitive decline. NFTs are mainly comprised of hyperphosphorylated tau, a protein particularly abundant in neurones, and lead to cell dysfunction and death (Theofilas et al., 2018). Compared with amyloid, there is less published evidence linking NFTs to VEGF-A biology. Nonetheless, diminished VEGF-A has been correlated with the presence of NFTs in AD cortices (Provias and Jeynes, 2008). Furthermore, the ratio of phosphorylated tau to amyloid is considered a strong positive predictor of cognitive decline and has been used to identify other CSF biomarkers for AD (Harari et al., 2014). A mouse model study demonstrated that lentiviral VEGF-A treatment can reverse AD-related increase in hyperphosphorylated tau as well as amyloid accumulation (Salomon-Zimri et al., 2016). Considering this evidence of crosstalk between amyloid, tau and VEGF-A, it is necessary to account for both amyloid and tau hypotheses in order to properly understand the role of VEGF-A in AD pathology.

It is increasingly clear that VEGF-A is not limited to its original discovery as a vascular protein but rather a pleiotropic protein that exerts cytoprotective effects in the nervous system (Greenberg and Jin, 2005) and the two families of isoforms—the VEGF-A_165_a and VEGF-A_165_b families (Harper and Bates, 2008) have different functions—while both can be neuroprotective (Beazley-Long et al., 2013), VEGF-A_165_b is not angiogenic and can block blood vessel growth induced by VEGF-A_165_a (Harper and Bates, 2008). It has thus been proposed that the VEGF-A_165_b isoforms act as homeostatic growth factors, whereas the VEGF-A_165_a are remodelling factors. As such VEGF isoforms have been proposed to act as a neuroprotective agent implicated in a number of neurodegenerative conditions, including AD (Storkebaum et al., 2004). In cases of ischemic injury, VEGF-A not only protects neural tissue by reperfusion but also directly exerts neurotrophic (survival) and neurotropic (neurogenesis) actions (Hobson et al., 2000). We therefore investigated the use of VEGF-A as a neuroprotective agent using *in vitro* models relevant to AD pathology. In addition, we aimed to look at the effects of the alternatively spliced isoform VEGF-A_165_b, which has previously been shown to protect against sensory neuronal degeneration in a model of diabetes (Hulse et al., 2015) but has not yet been studied in AD. The SRPK1 inhibitor Sphinx31, which can shift VEGF-A splicing from VEGF-A_165_a to VEGF-A_165_b isoforms, has never been used in the context of AD either. We initially established a neuronal cell assay based on oxidative stress since ROS production is widely accepted to be crucial to amyloid plaque toxicity (Cheignon et al., 2018), and then proceeded to use amyloid as a direct neurotoxin. Finally, we investigated the effect of VEGF-A and Sphinx31 treatments in an assay based on the induction of tau hyperphosphorylation (Metin-Armagan et al., 2018).

## 2. Materials and methods

### 2.1. Cell culture and cell differentiation

Unless otherwise stated, all cell culture reagents were purchased from Thermo Fisher Scientific. All cell culture was performed in cell culture hoods in class II facilities using aseptic technique and sterile culture medium. Cell culture flasks were kept in an incubator at 37°C in a humidified environment containing 5% CO_2_. SHSY5Y cells were cultured in DMEM/F-12 GlutaMAX media with 10% Foetal Bovine Serum (FBS); Neuro2a cells were cultured in RPMI media + 2 mM L-glutamine + 10% FBS.

Prior to treatment. SH-SY5Y cells were differentiated by reduction to 1% serum and treatment with 10 μM all-trans retinoic acid for 7 days. Differentiation media was renewed at least every 3 days. Neuro2a cells were differentiated by serum starvation and treatment with 300 μM dibutyryl cAMP for 48 h.

### 2.2. Cell viability assay

All cell viability assays were carried out on SHSY5Y cells and Neuro2a cells seeded in black-sided flat clear bottom 96-well plates. After differentiation and treatment, 10 μL WST-1 reagent was added to 100 μL media alone or cells with 100 μL treatment media. After 1 h, 2 h and (only if necessary) 4 h, plates were read at 450 nm with a reference wavelength of 620 nm. Incubations were performed under normal sterile cell culture conditions, at 37°C in a humidified environment containing 5% CO_2_. Readings from treated cells were normalised against an assay control (media + WST-1 reagent) and the relevant experimental control (e.g., vehicle only). Cells were treated with neurotoxic agents hydrogen peroxide, β-amyloid or okadaic acid for 24 h with and without 2.5 nM VEGF-A co-treatment. Neuroprotective agent NGF was used as a positive control. For SRPK1 inhibition, cells were treated with Sphinx31, synthesised as described by Batson et al. (2017) for 24–72 h (depending on assay) at a range of 1–10 μM.

### 2.3. Immunofluorescence

SHSY5Y cells were fixed in black-sided flat clear bottom 96-well plates with 4% PFA for 10 min at RT (50 μL per well). Fixed cells were washed once with 200 μL PBS and then permeabilised with 100 μL PBS 0.2% Triton X100 (PBSX) with 1% normal horse serum for 30 min. A mouse anti-human βIII tubulin antibody (R&D Systems) was used as a marker of neurite outgrowth. It was diluted 1:1,000 in 1% normal horse serum PBS and 50 μL was added to each well after cell permeabilisation, then incubated (in a humid box) overnight at 4°C. The following day, wells were washed three times for 5 min each with 200 μL PBS 0.5 mL/L Tween20. Donkey anti-mouse Alexafluor-488 secondary antibody and Hoechst were diluted 1:1,000 in 1% BSA PBS and 50 μL added to each well. Cells were incubated for 30 min in the dark at RT, and cells were washed again three times with 200 μL PBS 0.5 mL/L Tween20. Cells were kept in 100 μL PBS at 4°C until imaged. Fluorescence was captured using a Leica SPE confocal microscope: three 20x images were taken per well with smart gain of 700 or 800 (always kept consistent across experimental groups). There were 4–6 wells per condition in each experiment, making a total of 12–18 images per condition. Images were analysed on Image J using simple neurite tracer. The sum length of neurites per image was normalised to the number of cells, automatically calculated with a mask of Hoechst-stained nuclei. Average neurite length was then compared between treatment groups.

### 2.4. RNA extraction and amplification

#### 2.4.1. RNA extraction

RNA was extracted from cells after differentiation and treatment using a TRI-reagent protocol. Firstly, media was removed from cells and 500 μL TRI-reagent was added directly to culture dish. The cell lysate was homogenised with a pipette before transfer to an Eppendorf tube. After 5 min incubation at RT, 250 μL chloroform was added and samples were shaken vigorously by hand for 1 min, then left to incubate for 10 min at RT. Samples were centrifuged at 12,000 × *g* for 15 min at 4°C for phase separation: a red organic phase (containing protein), an interphase (containing DNA) and a colourless aqueous phase (containing RNA). The top aqueous phase was transferred into an ice-chilled Eppendorf tube with equal-volume isopropanol. Samples were shaken for 15 s and RNA was allowed to precipitate overnight at −20°C. Next, samples were centrifuged at 12,000 × *g* for 15 min at 4°C to pellet the RNA. The supernatant was discarded, and the RNA pellet was washed with 1 mL 75% ethanol and re-pelleted by centrifugation at 7,500 × *g* for 15 min at 4°C. The supernatant was discarded, and the RNA pellet was allowed to air-dry before being resuspended in 10–30 μL sterile water. RNA was kept on ice for 30 min before being quantified using a spectrophotometer (Thermo-Scientific Nanodrop 2000) and stored at −80°C.

#### 2.4.2. cDNA generation

RNA was reverse transcribed to complementary DNA (cDNA) using the Takara PrimeScript™ RT Reagent Kit (RR037A). For each sample, 1 μg RNA was combined with 25 pM oligo dT, 200 pM random hexamers and PrimeScript buffer, then made up to a reaction volume of 19 μL with sterile water. The reaction mixtures were heated to 65°C for 10 min for denaturation and then immediately placed on ice for the addition of 1 μL PrimeScript RT enzyme. Following this, reaction mixtures were incubated at 25°C for 10 min and 37°C for 60 min for generation of 50 ng cDNA. RT enzyme was inactivated by heating to 85°C for 1 min and cDNA samples were stored at -20°C. For each experiment, a no-template control was run to check for contamination, including all components of the reaction mixture excluding the RNA sample.

#### 2.4.3. Polymerase chain reaction

All PCR reactions were performed on 50 ng cDNA using GoTaq G2 Green Master Mix (Promega) at a total volume of either 15 or 30 μL. Primers were used at a final concentration of 0.4 μM. DNA samples were denatured at 96°C for 5 min, followed by multiple cycles of denaturation at 96°C for 30 s, annealing at primer specific temperature at 55°C for 30 s, and extension at 72°C for 1 min. PCR cycles were followed by a final extension step at 72°C for 10 min and samples were then held at 4°C until gel electrophoresis. PCR products were run in 2% agarose gels containing 50 ng/mL ethidium bromide at 100 V for approximately 1.5 h. Gels were visualised using the BioRad Gel Doc™ EZ System. Primers used were as follows: MAPT: Forward: 5′-CTCCAAAATCAGGGGATCGC-3′, Reverse 5′-CCTTGCTCAGGTCAACTGGT-3′. GAPDH Forward 5′-AATTC CATGGCACCGTCAAG-3′. Reverse 5′-GGTCATGAGTCCTTCCACGA-3′.

### 2.5. Protein extraction and assays

#### 2.5.1. Cell lysis

Protein was extracted from cells after differentiation and treatment. Cells were washed with ice-cold PBS and kept on ice. Cell lysis buffer (NP40, 1X, PMSF, 1 mM, Na_3_VO_4_, 10 mM, NaF, 5–50 mM Protease Inhibitor, 1X Roche complete cocktail) was added to cells and left to incubate for 10 min. Cells adhered to the surface of culture plates were scraped and mechanically dissociated by trituration, and then transferred to an ice-chilled Eppendorf. The cell suspension was vortexed for 15 s three times over a 10 min incubation on ice. Samples were centrifuged at 12,000 × g for 10 min at 4°C and the supernatant was collected in a fresh Eppendorf and stored at −80°C.

#### 2.5.2. Protein quantification

A Bradford protein reagent assay (Bio-Rad) was used to quantify the protein concentration of samples against a seven-point standard curve of bovine serum albumin (BSA) in PBS (1,000 μg/mL serially diluted 1:1 to 15.625 μg/mL). Samples were diluted 1:10 or 1:20 in PBS to bring them within range of the standard curve. 10 μL of standards and samples were loaded in triplicate into a clear 96 well plate. Bradford reagent was diluted 1:5 in distilled water and 200 μL was loaded to each well. Plates were agitated for 5 s and then read immediately at 620 nm using a Magellan microplate reader. A standard curve was plotted on Microsoft Excel using optical density readings from the BSA standards, and sample protein concentrations were calculated based on the equation of the curve.

#### 2.5.3. ELISA

An antibody selective for the VEGF-A_165_a isoform was generated by BioRad HuCAL technology using a peptide corresponding to the c terminus of VEGF-A_165_a (TCRCDKPRR). High-binding 96-well clear microplates were coated with 100 μL capture antibody at 075 μg/mL (anti-VEGF-A_165_a) or 10 μg/mL (anti-VEGF-A_165_b, MRVL56/1, Abcam), sealed with parafilm and incubated (with agitation) overnight at RT. The following day, wells were washed three times with wash buffer (PBS + 0.05% Tween20). After each wash, plates were inverted and firmly tapped on tissue paper to completely empty wells and prevent carry-over of liquid or bubbles. 100 μL blocking buffer [PBS + 1% BSA (sterile-filtered) or SuperBlock (ThermoFisher)] was added to coated plates, sealed with parafilm, and incubated (with agitation) for 2 h at RT. After block, wells were washed again (as before) and 100 μL standards or samples were added in duplicate according to plate layout planned for the experiment. VEGF-A_165_a and VEGF-A_165_b recombinant proteins were used as standards, starting at 1000 pg./mL and serially diluted 1:1 in diluent to a low concentration of 3.9 pg./mL, making a nine-point standard curve. The opposite VEGF-A isoform was used at highest concentration as a negative control to ensure specificity of the capture antibody. Samples were typically diluted 1 in 2, also using diluent [PBS + 1% BSA (sterile-filtered)]. After addition of standards and samples, plate was resealed with parafilm and incubated (with agitation) for 2 h at RT. As detection antibody, 100 μL biotinylated mouse monoclonal anti-human VEGF-A antibody (BAF293, R&D Systems) was added to each well following another set of washes. Plates were resealed and incubated (with agitation) for another 2 h at RT. Plates were washed (as before) and 100 μL horseradish peroxidase conjugated streptavidin (made up in diluent) was then added to each well. Plates were sealed with foil to protect from light and incubated for 30 min at RT. Plates were washed a final time and 100 μL tetramethylbenzidine (TMB) substrate was added to each well, then incubated in the dark for 1 h (or until the colour change reached an appropriate intensity) at RT. The reaction was quenched with 50 μL 1 M HCL per well and shaking for 10 s to ensure thorough mixing. Finally, plates were read at 450 nm using a Magellan microplate reader. A standard curve was plotted on Microsoft Excel using optical density readings from the VEGF-A standards, and sample concentrations for VEGF-A isoforms were calculated based on the equation of the curve.

### 2.6. Statistical analysis

Statistics were performed in Graphpad Prism. Curve fitting was undertaken with a four variable curve fit. Statistical tests were undertaken as described in the figures. *Post hoc* analysis was undertaken for one-way ANOVA using Holm-Sidak tests. Values are given as mean ± standard error of the mean unless otherwise stated. Ratios of VEGF-A_165_a to VEGF-A_165_b were calculated as the concentration of VEGF-A_165_a to that of VEGF-A_165_b.

## 3. Results

### 3.1. VEGF-A isoforms are neuroprotective against oxidative stress

**Figure 1:**
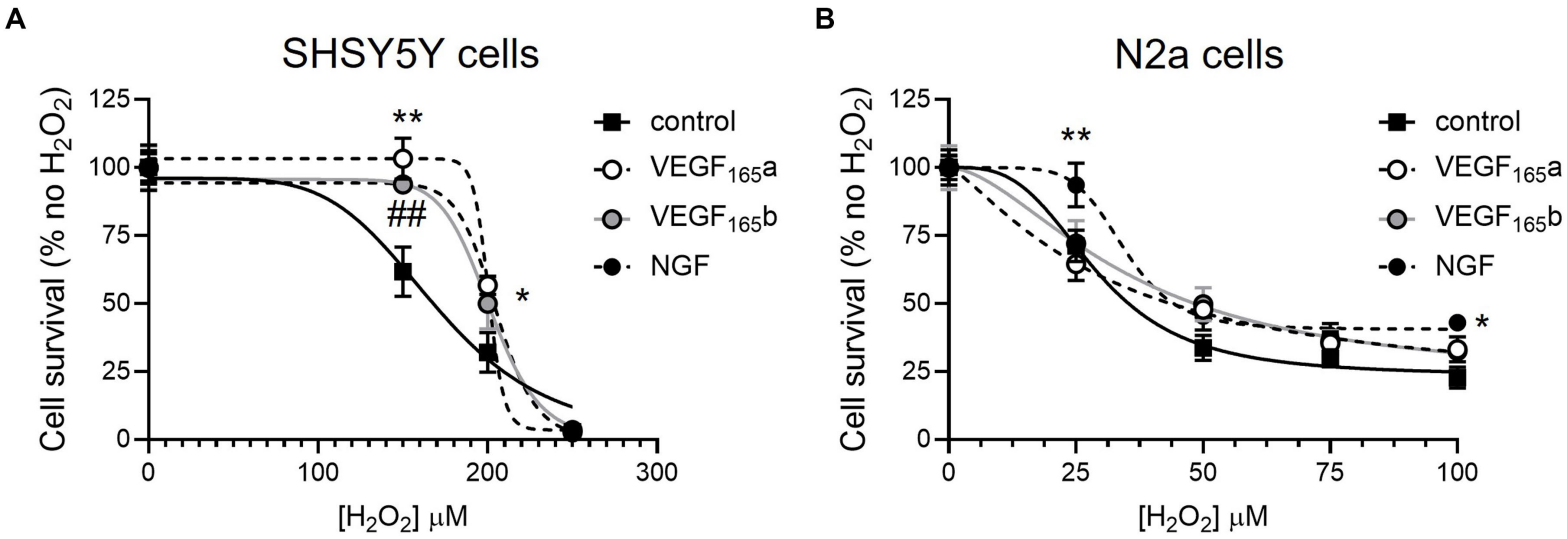
VEGF-A isoforms increase metabolic activity of SHSY5Y cells but not N2a cells co-treated with hydrogen peroxide. SHSY5Y cells and N2a cells were treated with H_2_O_2_ alone or with NGF or 2.5 nM VEGF-A isoforms for 24 h, and a WST-1 assay was performed to measure metabolic activity. Readings were normalized against an assay control (media + WST-1 reagent) and experimental control (cells treated with PBS or NGF alone + WST-1 reagent). **(A)** Concentration-dependent decrease in percent metabolic activity of SHSY5Y cells with H_2_O_2_ without (control) or with NGF, VEGF-A_165_a, or VEGF-A_165_b (two-way ANOVA, *p* < 0.01). **(B)** Concentration-dependent decrease in percent metabolic activity of N2a cells with H_2_O_2_ without (control) or with NGF, VEGF-A_165_a, or VEGF-A_165_b (two-way ANOVA, *p* < 0.0001). *N* = 8 or *N* = 12 readings. ^*^VEGF-A_165_a different from control; ^##^VEGF-A_165_b different from control; and ^*^NGF different from control. ^*^p < 0.05, ^##, **^p < 0.01, compared with control, Holm Sidak *post hoc* tests. *N* = 12 per group per concentration.

To establish an assay of neuronal toxicity, SHSY5Y and N2a cells were treated with hydrogen peroxide, a strong reactive oxygen species that can induce oxidative stress *in vitro* (Bonello et al., 2007) and has been shown in other cell types to be modified by VEGF isoforms, and therefore serves as a good positive control (Beazley-Long et al., 2013). The WST-1 assay showed a decreased cell viability at 150–250 μM in SHSY5Y cells (Figure 1A) and 25–100 μM H_2_O_2_ in N2a cells (Figure 1B). Co-treatment with the neuroprotective agent NGF was used as a positive assay control since it is known to consistently increase neuronal cell survival (Bogetti et al., 2018). In the WST-1 assay, NGF resulted in a significant increase in viability of both cell lines (two-way ANOVA, *p* < 0.01 and *p* < 0.0001 respectively), validating the use of the assay to investigate the effect of VEGF-A isoforms in this *in vitro* model of oxidative stress. In SHSY5Y cells, treatment with 2.5 nM recombinant VEGF-A_165_a or VEGF-A_165_b significantly increased cell viability (two-way ANOVA, *p* < 0.01) in a similar manner to NGF (Figure 1A). There was an upward shift in cell metabolic activity where the drop in the control group induced by 150 μM H_2_O_2_ (62 ± 9.8% of control) was completely recovered by VEGF-A_165_a (103 ± 7.5% of no H_2_O_2_) and VEGF-A_165_b (93.8 ± 2.5, shown in Figure 1A). In N2a cells, there was no significant difference in the viability with VEGF-A treatment of either isoform (see Figure 1B).

### 3.2. VEGF-A isoforms are neuroprotective against Aβ-induced neurotoxicity

**Figure 2:**
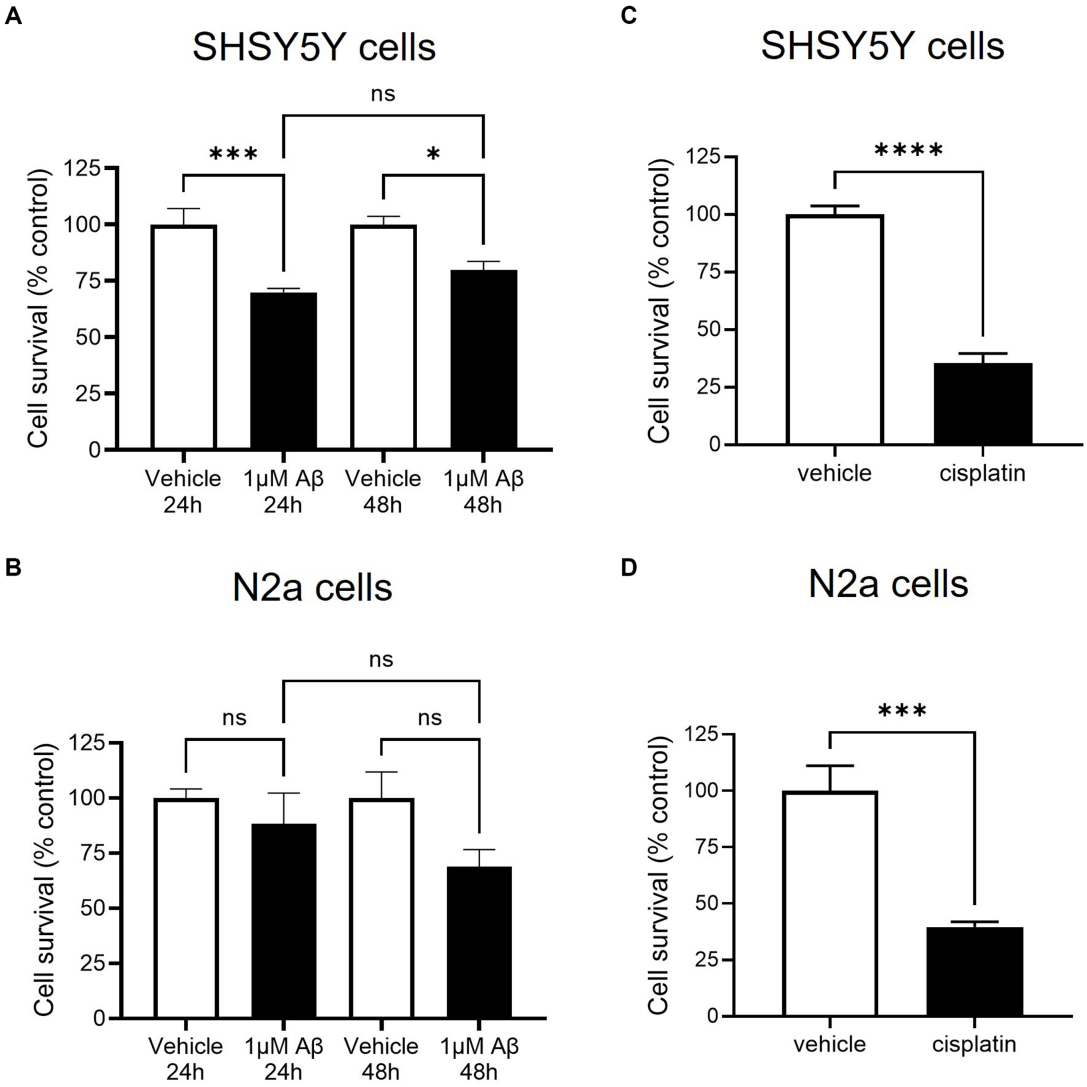
β-amyloid (Aβ) decreased cell survival as measured by metabolic activity of SHSY5Y cells but not in N2a cells. SHSY5Y cells and N2a cells were treated with 1 μM Aβ for 24 and 48 h. WST-1 assay was performed to measure metabolic activity and readings were normalised against an assay control (media + WST-1 reagent) and experimental control (cells treated with vehicle alone + WST-1 reagent). *N* = 6 readings. **(A)** After both 24 and 48 h treatment, SHSY5Y cell metabolic activity was significantly decreased with 1 μM Aβ (one-way ANOVA, *p* < 0.001). **(B)** N2a cells had a variable response to Aβ; metabolic activity was reduced but not to a significant degree (one-way ANOVA, *p* > 0.05). **(C,D)** To confirm the assay worked, cisplatin treatment was shown to decrease metabolic activity in both SHSY5Y cells and N2a cells. ns, not significant; ^*^p < 0.05, ^***^p < 0.001; ^****^p < 0.0001 compared with vehicle, Holm Sidak *post hoc* test.

To model amyloid cytotoxicity in AD, Aβ peptide treatment was used to reduce cell viability in accordance to guidance from previously published methods to prepare amyloid in unaggregated, oligomeric and fibrillar states (Stine et al., 2011). Since amyloid has a tendency to aggregate *in vitro* and its unaggregated form had been shown to reduce viability of N2a cells, this method was selected. In SHSY5Y cells 1 μM amyloid significantly decreased viability to 55% of control after 24 h (Figure 2A). Since 48 h treatment produced a very similar response, the remainder of assays were carried out after 24 h incubation. The same optimisation was repeated in N2a cells: although viability decreased to 69 ± 8% of control after 48 h 1 μM amyloid treatment, the assay did not produce a statistically significant decrease in viability (see Figure 2B). To validate the use of WST-1 as a measure of cell viability, both SHSY5Y and N2a cells were treated with a positive control chemotherapy agent cisplatin: 24 h treatment produced a decrease to 35 ± 4 and 40% ±2% of control viability, shown in Figures 2C,D, respectively.

**Figure 3:**
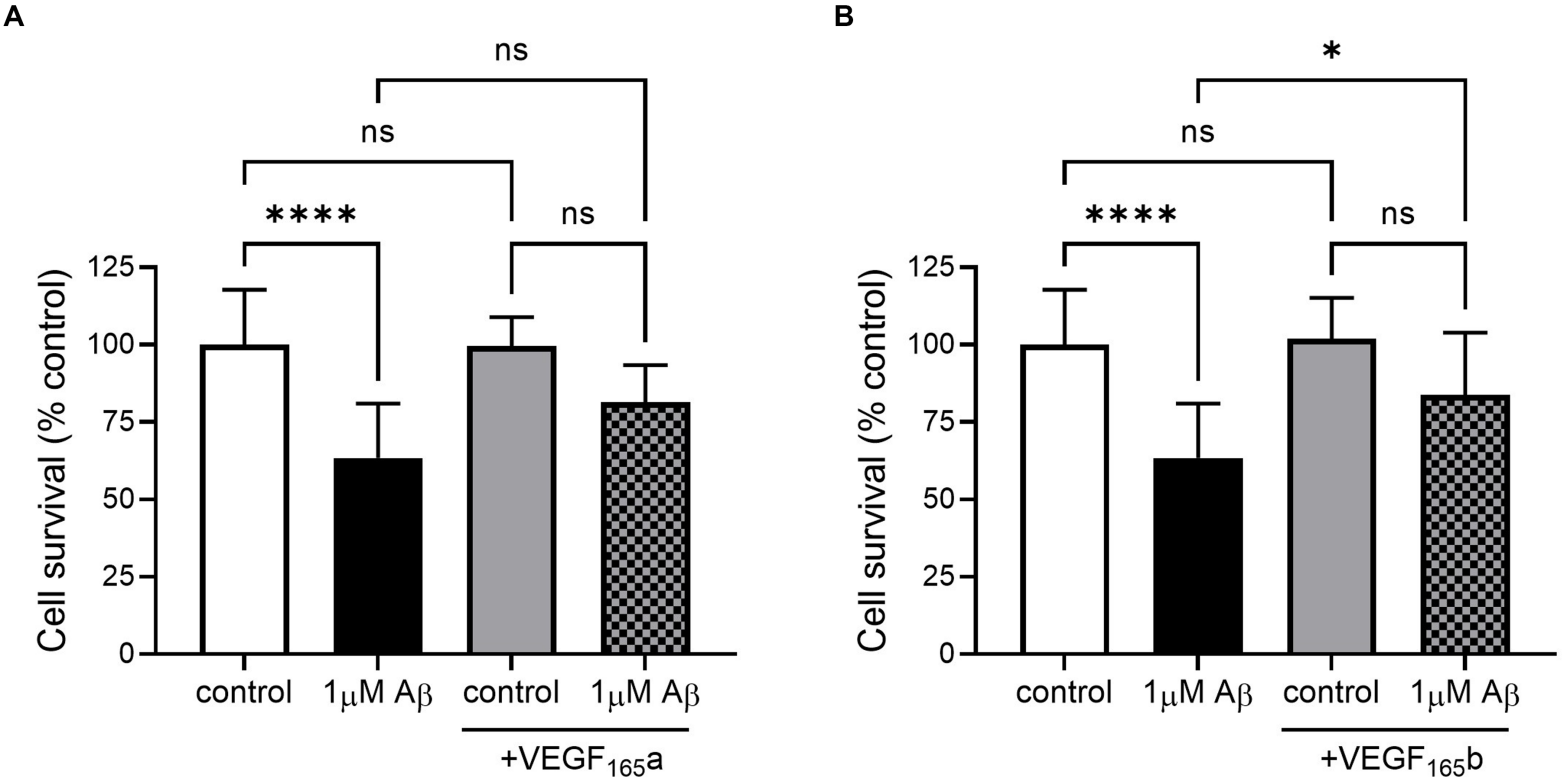
VEGF-A_165_a or VEGF-A_165_b treatment recovers Aβ-induced decrease in metabolic activity in SHSY5Y cells. **(A)** Normalised metabolic activity showed decrease with 1 μM Aβ (one-way ANOVA, *p* < 0.0001) and was recovered with co-treatment of 2.5 nM VEGF-A_165_a. Treatment with 2.5 nM VEGF-A_165_a alone did not significantly change metabolic activity. **(B)** Similarly, 2.5 nM VEGF-A_165_b alone did not change metabolic activity but protected against Aβ-induced reduction. *N* = 4–6. ^*^p < 0.05, ^****^p < 0.0001 compared with vehicle, Holm Sidak *post hoc* tests.

In this model, SHSY5Y cells were treated with recombinant VEGF-A_165_a and VEGF-A_165_b to identify whether they could rescue amyloid-related decrease in cell viability. Treatment with 1 μM amyloid alone significantly decreased cell viability, but when co-treated with either VEGF-A_165_a (in Figure 3A) or VEGF-A_165_b (in Figure 3B), cell viability returned towards control levels.

### 3.3. VEGF-A isoforms are neuroprotective against tau hyperphosphorylation induced cytoskeletal destabilisation

**Figure 4:**
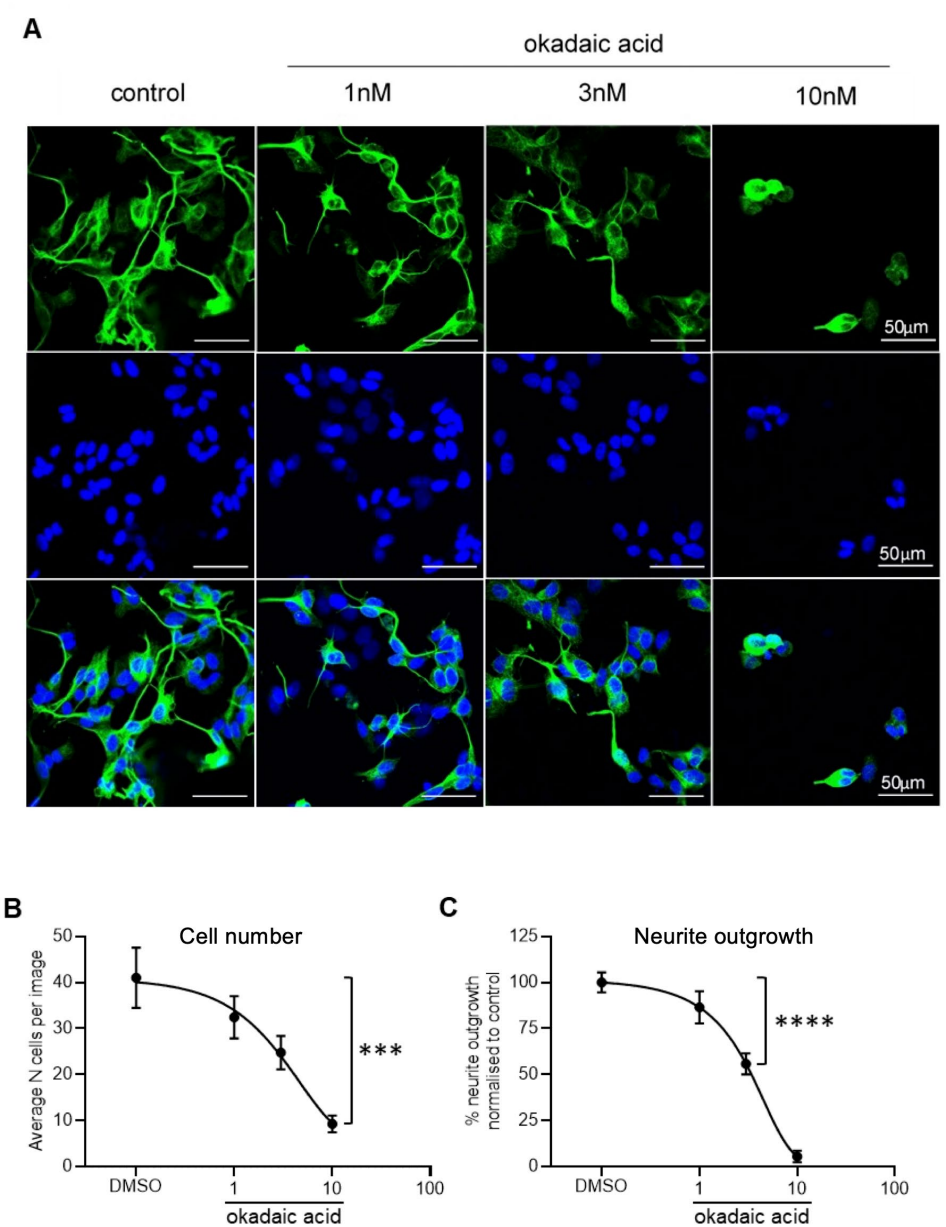
Reduced neurite outgrowth with increased concentration of OA. **(A)** Neuron-specific marker βIII tubulin identified neurites in SHSY5Y cells shown in green. Cell nuclei stained with Hoechst shown in blue. Merged images in final row. *N* = 18 images per condition. **(B)** Decreased number of live cells with increased concentration of OA. Nuclei automatically counted on FIJI software with a macro. **(C)** Neurite length was quantified using simple tracer and the sum neurite length was divided by number of nuclei. The outgrowth in SHSY5Y cells treated with OA was plotted as percentage of DMSO control. Points = mean, error bars = SEM. ^****^p < 0.0001 compared with vehicle. One way ANOVA with Holm Sidak *post hoc* tests. *N* = 3 with six images analysed per repeat.

To model tau hyperphosphorylation toxicity in AD, neurite outgrowth was selected as a basic measure of neuronal viability and function. This assay is closely related to the primary role of tau as a microtubule-associated protein. Tau stabilises the neuronal cytoskeleton by copolymerising with tubulin for microtubule formation. However, an increase in its phosphorylation reduces its interaction with microtubules, causing destabilisation of the cytoskeleton and neurodegeneration (Lindwall and Cole, 1984; Iqbal et al., 2010). βIII tubulin antibody was selected as a neuronal specific marker (Draberova et al., 1998) and used to probe SHSY5Y cells treated with increasing concentrations of okadaic acid (OA), as presented in Figure 4A. OA was used a neurotoxic agent which induces hyperphosphorlyation of tau (Martin et al., 2011). Quantification showed a concentration-dependent decrease in cell number (Figure 4B) and normalised neurite outgrowth length (Figure 4C). At 3 nM OA, SHSY5Y cells decreased to 60 ± 9% of control cell number and 56 ± 9% of control neurite outgrowth length.

**Figure 5:**
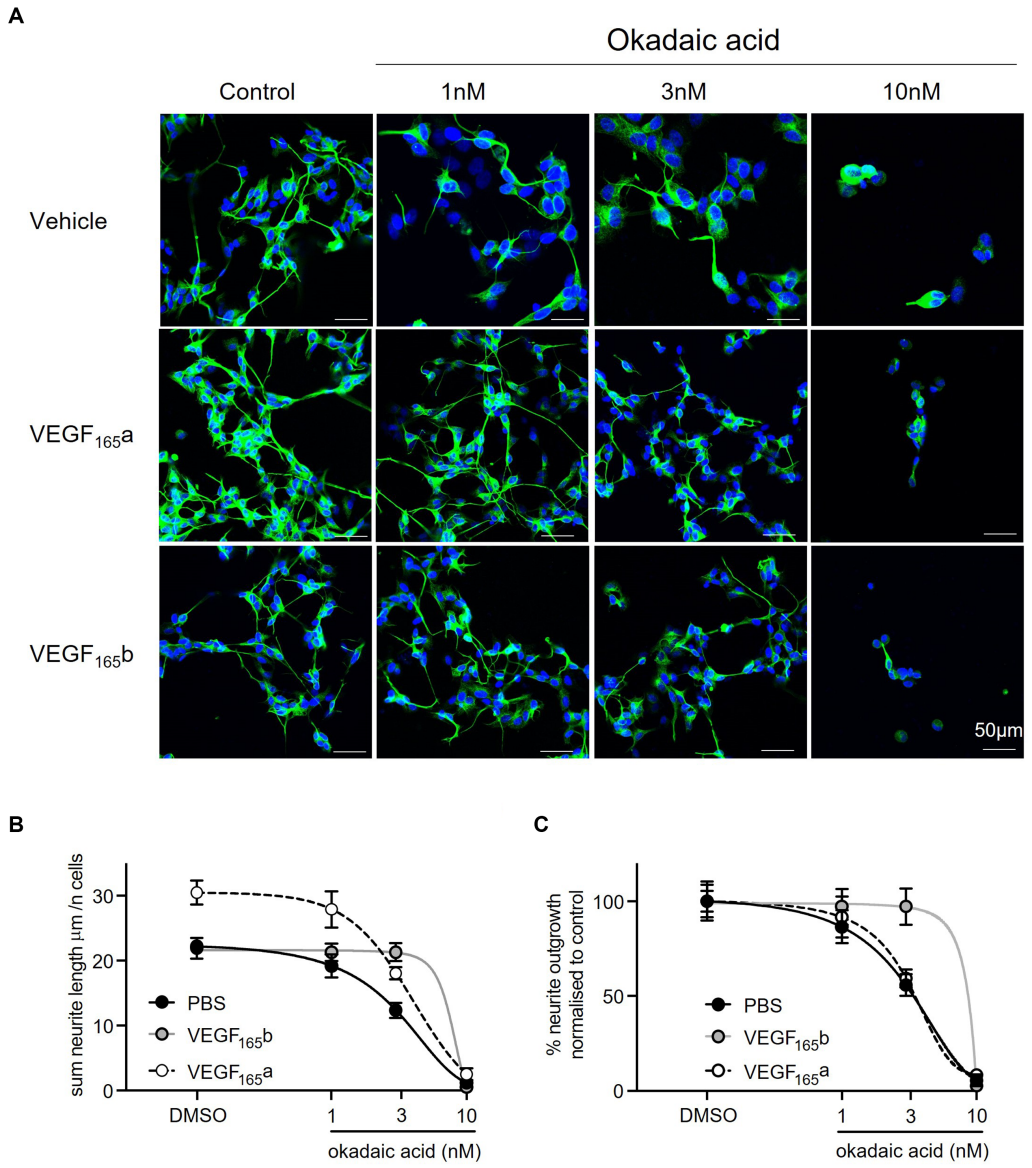
VEGF-A_165_a increased neurite outgrowth in SHSY5Y cells co-treated with OA. **(A)** neuron-specific marker βIII tubulin identified neurites in green. Cell nuclei stained with Hoechst shown in blue. *N* = 18 images per condition. **(B)** Using FIJI software, neurite length was quantified using simple tracer, and the sum neurite length was divided by number of nuclei counted with a macro. Plot showed that average neurite length is significantly and consistently higher with VEGF-A_165_ in control, 1 and 3 nM OA treatments (two-way ANOVA, *p* < 0.0001). With 10 nM OA, VEGF-A_165_ did not have an effect. By itself, VEGF-A_165_b did not increase average neurite length but maintained outgrowth in SHSY5Y cells co-treated with 1 and 3 nM OA where average length was significantly higher than PBS control (two-way ANOVA, *p* < 0.0001). With 10 nM OA, VEGF-A_165_b did not have an effect. **(C)** Neurite outgrowth was plotted as percentage of DMSO control with and without VEGF-A_165_ treatment. Points = mean, error bars = SEM. ^*^p < 0.05 compared with PBS, ^**^p < 0.01 compared with PBS, ^***^p < 0.001 compared with PBS, ^###^*p* < 0.001 compared with VEGF-A_165_b. Two way ANOVA with Holm Sidak *post hoc* tests. *N* = 3 with six images analysed per repeat.

SHSY5Y cells co-treated with OA and recombinant VEGF-A_165_a (as shown in the second panel of Figure 5A) had significantly increased neurite outgrowth compared with vehicle and with OA alone (two-way ANOVA, *p* < 0.0001). There is an upward shift in neurite length, where in the absence of OA, the average neurite length per cell increased from 22 ± 1 to 30 ± 2 μm (see Figure 5B). With up to 3 nM OA, VEGF-A_165_a maintained higher neurite outgrowth in SHSY5Y cells, as seen by the increase from 12 ± 1 to 18 ± 1 μm. When neurite outgrowth was normalised to the -OA control in each group, there was overlap between two curves with and without VEGF-A_165_a (see Figure 5C), indicating that VEGF-A_165_a increased baseline neurite outgrowth but did not counteract the OA-induced decrease. For example, with 3 nM OA, PBS and VEGF-A_165_a treated cells had an average 56 ± 5 and 59 ± 3% of their -OA controls, respectively.

In comparison, recombinant VEGF-A_165_b alone did not induce an increase in neurite outgrowth in vehicle treated SHSY5Y cells (as shown in the second panel of Figure 5A). However, VEGF-A_165_b treated SHSY5Y cells did maintain significantly higher sum neurite outgrowth when treated with OA (two-way ANOVA, *p* < 0.0001). While 3 nM OA decreased neurite outgrowth from 22 ± 1 to 12 ± 1 μm in the control group, VEGF-A_165_b treated cells had average neurite length of 22 ± 2 and 21 ± 1 μm, respectively (see Figure 5B). Maintaining similar neurite outgrowth by VEGF-A_165_b treatment resulted in a rightward shift in the normalised curve, as shown in Figure 5C.

To quantify the effect of VEGF-A isoforms on OA-induced neurite “dieback,” the half maximal inhibitory concentration (IC_50_) of OA on sum outgrowth (per cell) was compared between groups co-treated with vehicle only, VEGF-A_165_a or VEGF-A_165_b (Table 1). VEGF-A_165_a treatment caused a modest increase in IC_50_ from 3.0 to 3.4 μM whilst VEGF-A_165_b treatment caused a greater increase to 5.1 μM. This suggests that VEGF-A_165_b treatment exerts a stronger protective effect on outgrowth as higher concentration of OA is required to produce the same level of neurite “dieback.”

**Table 1:** Summary table for the effect size and IC_50_ of VEGF-A isoforms on OA-induced neurite “dieback” in SHSY5Y cells.

Co-treatment	PBS(vehicle)	VEGF-A_165_a	VEGF-A_165_b
Bottom	Constrained> = 0		
Top	24.18	33.36	24.83
LogIC50	0.479	0.534	0.710
IC50	3.011	3.423	5.133

Values based on *N* = 18 images per condition and quantification of sum neurite outgrowth normalised to the number of cells in field of view.

### 3.4. SRPK1 inhibition can change splicing of VEGF-A in SHSY5Y cells

**Figure 6:**
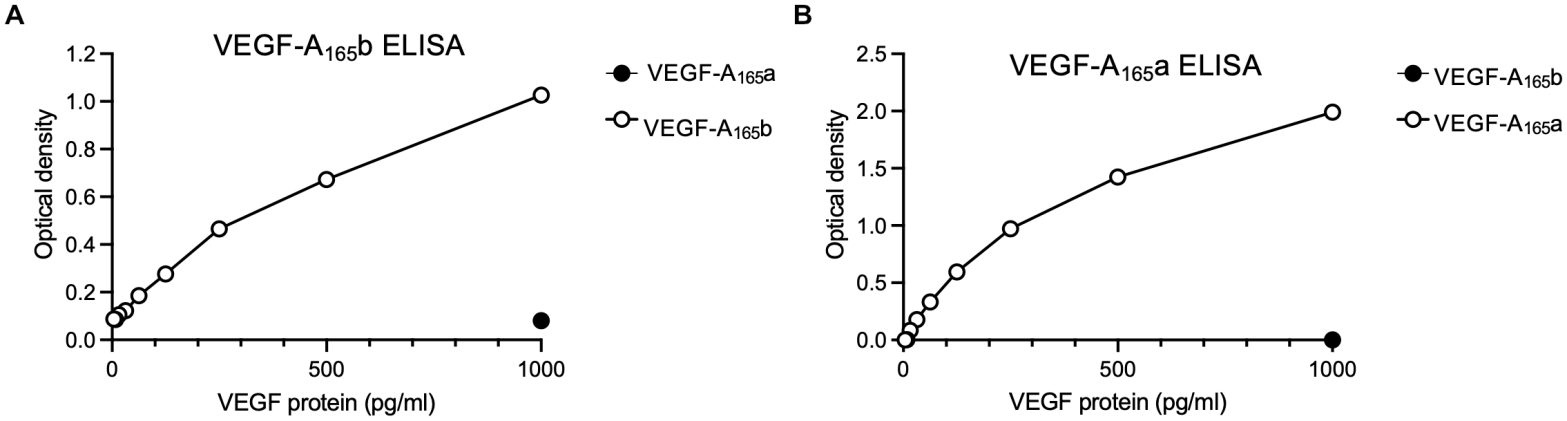
Standard curves for the **(A)** VEGF-A_165_b ELISA and **(B)** VEGF-A_165_a ELISA, which show detection window for protein expression. Points on each graph are average of two independent ELISA plates, run for SHSY5Y cell lysates and media samples.

Sphinx31 is a SRPK1 inhibitor that has been shown to switch splicing from VEGF-A_165_a to VEGF-A_165_b in epithelial cells, as well as dorsal root ganglionic cells. Consequentially, Sphinx31 is a powerful investigatory tool for alternative splicing of VEGF-A and other genes regulated by SRPK1. However, Sphinx31 has not been previously used on SHSY5Y cells. Since recombinant VEGF-A_165_b was shown to have neuroprotective effects in SHSY5Y cells, we aimed to determine whether use of Sphinx31 could increase endogenous expression of VEGF-A_165_b, and potentially replicate the same physiological results. To quantify relative expression of VEGF-A isoforms, we used a highly sensitive measure of expression, ELISA, to detect relative expression of VEGF-A_165_a and VEGF-A_165_b isoforms in SHSY5Y cells. Optimisation of this ELISA is described in the methods, and the standard curves used for relative protein quantification are shown in Figure 6 (minus “blank” i.e., OD measured from 1% BSA only). All values were then normalised to vehicle control group VEGF-A isoform expression.

**Figure 7:**
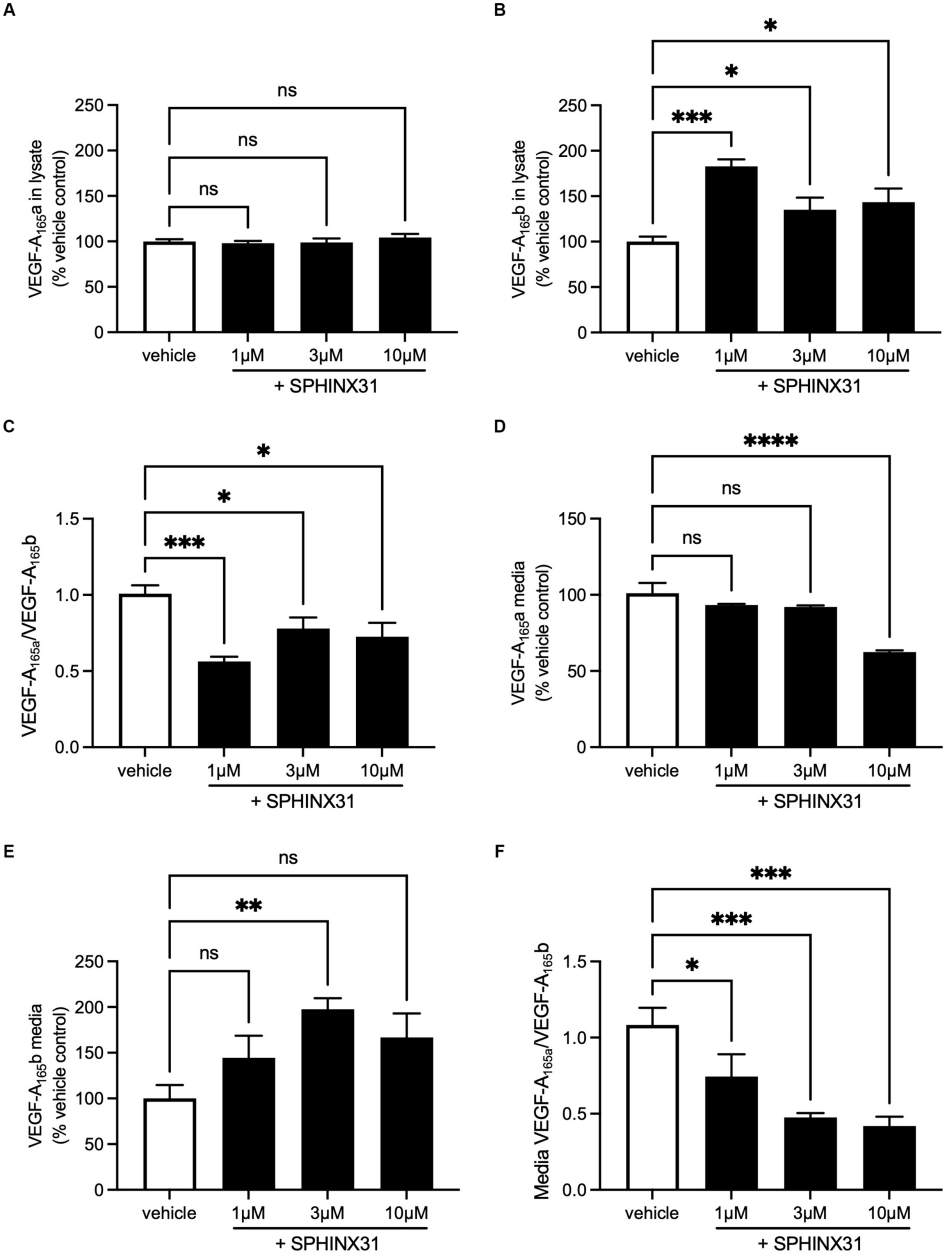
Sphinx31 treatment significantly reduces VEGF-A_165_a:VEGF-A_165_b ratio expression in SHSY5Y cells. **(A)** Sphinx31 does not produce measurable change in VEGF-A_165_a expression in cell lysate. **(B)** Cell lysate shows a significant increase in VEGF-A_165_b Sphinx31 (*p* < 0.001). **(C)** The ratio of VEGF-A_165_a:VEGF-A_165_b is significantly reduced in cell lysate with Sphinx31 treatment, but this does not occur in a concentration-dependent manner (*p* < 0.001 one-way ANOVA). **(D)** VEGF-A_165_a in cell media remains stable with 1–3 μM Sphinx31 but is significantly reduced with 10 μM treatment (*p* < 0.0001, one-way ANOVA). **(E)** There was an increase in VEGF-A_165_b in the media with Sphinx31 treatment. Measured increase with 3 μM is statistically significant (*p* < 0.05, one-way ANOVA). **(F)** The ratio of VEGF-A_165_a:VEGF-A_165_b is dose dependently reduced in media after treatment with Sphinx31 (*p* < 0.001 and *p* < 0.05, one-way ANOVA). *N* = 3 biological replicates and *N* = 2 technical replicates.

Expression of both isoforms was detectable in both cell lysate and media collected from SHSY5Y samples. In the cell lysate, VEGF-A_165_a expression did not change with Sphinx31 treatment (Figure 7A) but VEGF-A_165_b significantly increased 82.6 ± 7.7% above vehicle control with 1 μM Sphinx31 (Figure 7B, one-way ANOVA, *p* < 0.001). The ratio of VEGF-A_165_a:VEGF-A_165_b in cell lysate was calculated to show a significant decrease with both 1 and 10 μM Sphinx31 treatment (shown in Figure 7C, *p* < 0.001 and *p* < 0.05, respectively). However, this effect was not concentration-dependent, suggesting that the lowest concentration 1 μM Sphinx31 was sufficient to induce a shift in VEGF-A splicing towards the VEGF-A_165_b isoform, and this was the maximal effect observable in SHSY5Y cells through SRPK1 inhibition.

In cell media, VEGF-A_165_a expression significantly reduced with 10 μM Sphinx31 treatment to 63.5% ± 1.1% of vehicle control, as shown in Figure 7D (*p* < 0.0001, one-way ANOVA). In contrast, VEGF-A_165_b expression showed a general increase, albeit variable (Figure 7E). At 3 and 10 μM Sphinx31, VEGF-A_165_b expression was measured to be 97.3 ± 12.2% and 66.6 ± 26.1% higher than vehicle control, respectively (*p* < 0.05 at 3 μM treatment, one-way ANOVA). This caused a significant decrease in the ratio of VEGF-A_165_a:VEGF-A_165_b in cell media at both 3 and 10 μM Sphinx31 treatment (Figure 7F). As a measure of secreted protein, this confirms that altered splicing of VEGF-A in Sphinx31-treated SHSY5Y cells can be quantified in both intracellular and extracellular protein.

### 3.5. SRPK1 inhibition is neuroprotective against tau hyperphosphorylation

**Figure 8:**
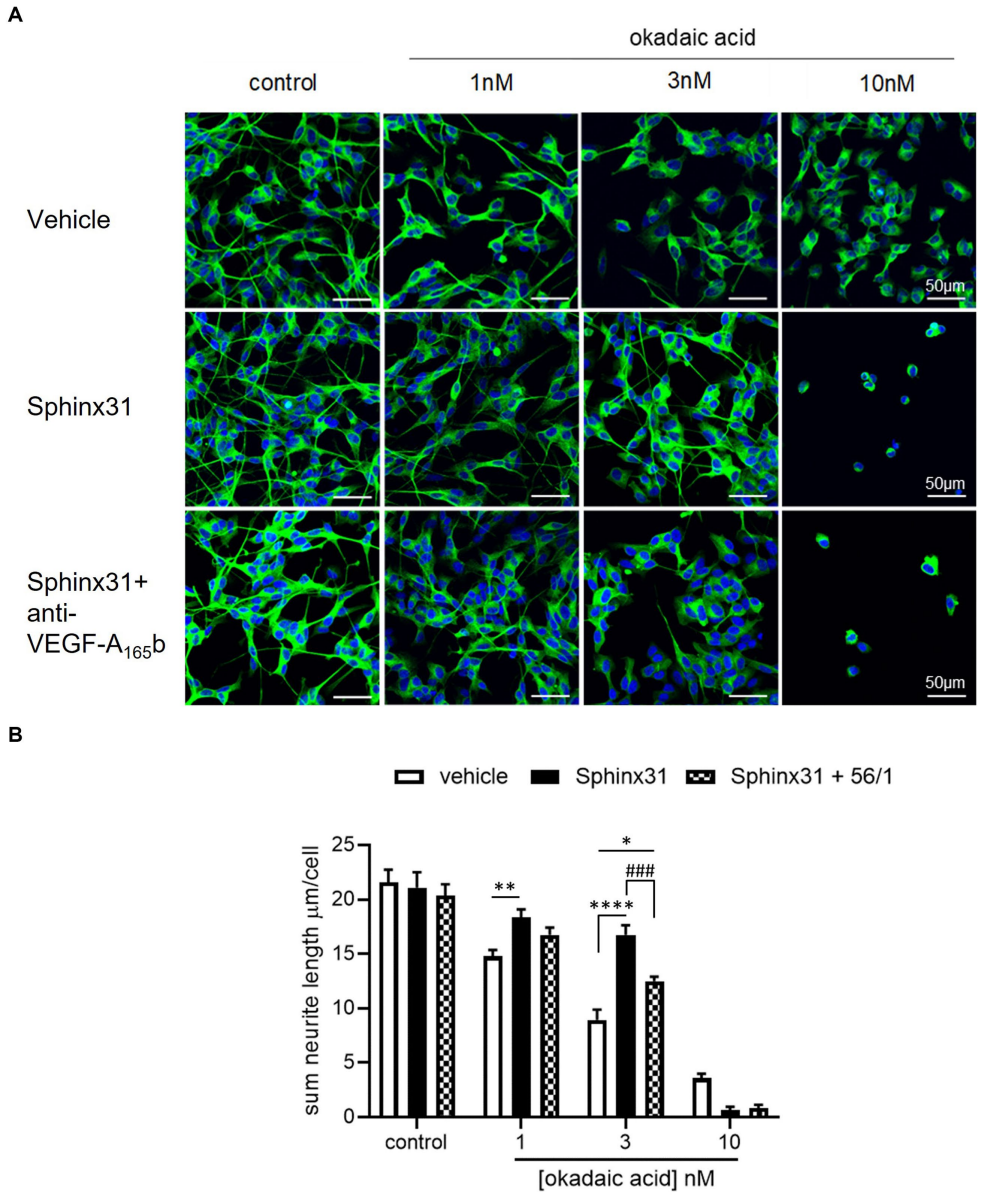
Sphinx31 significantly ameliorates OA-induced decline in neurite outgrowth from SHSY5Y cells. **(A)** neuron-specific marker βIII tubulin identified neurites in green. Cell nuclei stained with Hoechst shown in blue. Neurite outgrowth was calculated by tracing neurites on Image J and dividing the sum length per image by the number of cells. The top row of images shows OA vehicle alone, the middle row shows co-treatment with Sphinx31, the bottom row treatment with Sphinx31 and anti-VEGF-A_165_b. **(B)** Sphinx31 co-treatment significantly ameliorates decline in neurite outgrowth induced by 1 and 3 nM OA, which was inhibited by treatment with anti-VEGF-A_165_b (two-way ANOVA, *p* < 0.01 and *p* < 0.0001, respectively). *N* = 2 with six images analysed per repeat. Error bars = SEM.

Based on the findings that VEGF-A_165_b can increase outgrowth of SHSY5Y cells and Sphinx31 can shift splicing from VEGF-A_165_a towards VEGF-A_165_b, we determined whether 3 μM Sphinx31 treatment would have the same effects on neurite outgrowth as recombinant VEGF-A_165_b. Using the OA neurotoxicity model (with DMSO vehicle control, representative images shown in Figure 8A), Sphinx31 treatment protected against OA-induced decline in SHSY5Y cell outgrowth. As shown in Figure 8B, OA significantly reduces outgrowth in a concentration-dependent manner, while Sphinx31 ameliorates OA-induced neurite “dieback.” At 1 nM OA, Sphinx31-treated cells showed increased neurite outgrowth from an average of 15 ± 1 to 28 ± 1 μm (two-way ANOVA, *p* < 0.01). Similarly, at 3 nM OA, Sphinx31 treatment increased outgrowth from an average of 9 ± 1 to 17 ± 1 μm (two-way ANOVA, *p* < 0.0001). At 10 nM OA, Sphinx31 treatment was no longer neuroprotective. In fact, outgrowth was effectively ablated with this treatment combination, as very few cells were observed compared to the control group (Figure 8A). Similar results were seen in previous iterations of this assay with PBS vehicle control, which could imply that DMSO alone may affect outgrowth.

#### 3.5.1. SRPK1 inhibition is neuroprotective through a VEGF-A_165_b mediated mechanism

To determine whether the neuroprotective effect of Sphinx31 is dependent on alternative splicing of VEGF-A, the outgrowth assay was repeated with a VEGF-A_165_b neutralising antibody co-treatment (Figure 8B). Compared to Sphinx31 alone, anti-VEGF-A_165_b-treated SHSY5Y cells showed significantly reduced outgrowth (Figure 8B), indicating that amelioration of outgrowth with Sphinx31 does in fact occur, at least partially, through increased VEGF-A_165_b expression. In the presence of 1 and 3 nM OA respectively, when co-treated with both Sphinx31 and anti-VEGF-A_165_b, there was a significant reduction in neurite outgrowth compared to Sphinx31 alone, at an average sum length of 13 ± 0.5 μm (two-way ANOVA, *p* < 0.001, Figure 8B). However, this remained significantly higher than the control group at 3 nM OA (two-way ANOVA, *p* < 0.05). It is possible that (a) anti-VEGF-A_165_b treatment did not completely ablate VEGF-A_165_b at concentration 1 ng/mL, or (b) Sphinx31 is neuroprotective through other mechanisms in addition to VEGF-A alternative splicing.

#### 3.5.2. OA and Sphinx31 treatments do not significantly alter the 4R:3R tau ratio in SHSY5Y cells

**Figure 9:**
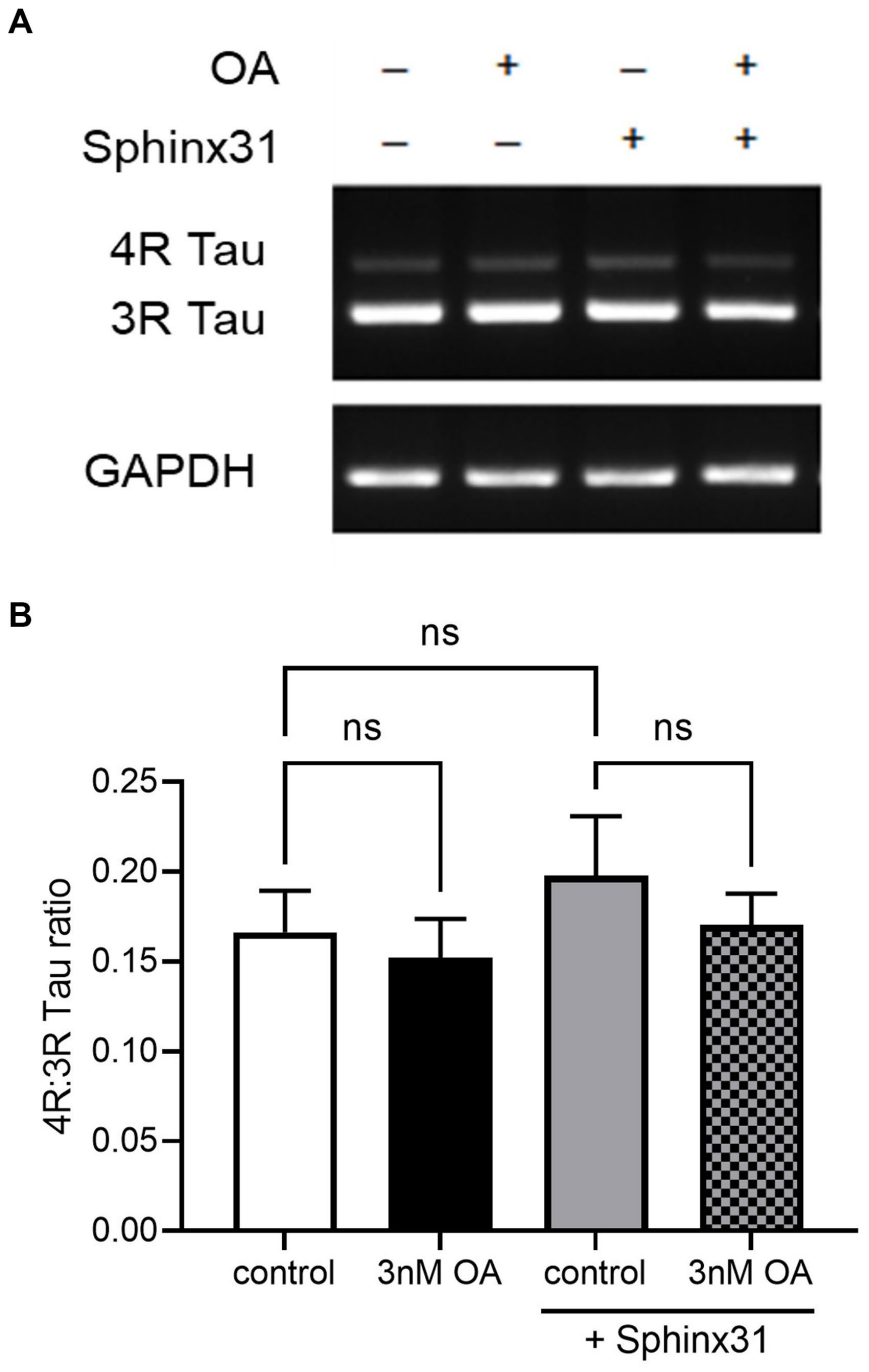
OA and Sphinx31 treatments do not significantly alter the 4R:3R tau ratio in SHSY5Y cells. **(A)** SHSY5Y cells were treated with 3 nM OA and/or 3 μM Sphinx31 for 24 h. *MAPT* PCR was carried out using primers spanning exon 10 (forward primer in exon 9, reverse primer in exon 11) to quantify relative expression of 4R and 3R tau isoforms. Images show tau isoform expression and housekeeping gene *GAPDH* matched to each sample. **(B)** Sphinx31 treatment does not significantly alter 4R:3R tau ratio (one-way ANOVA, *p* > 0.05). *N* = 4, error bars show SEM.

Okadaic acid was used here to model AD-related neurotoxicity, since OA is known to induce hyperphosphorylation of tau (Martin et al., 2011). However, the relative expression of 4R and 3R tau isoforms is also important to AD pathology (Espindola et al., 2018). Since SRPK1 is a splicing kinase, and Sphinx31 is a selective and potent SRPK1 inhibitor, we determined whether Sphinx31 and OA co-treatment caused a shift in the 4R:3R tau ratio. Note that although SHSY5Y cells preferentially express 3R tau, they do endogenously express both 4R and 3R tau isoforms. Sphinx31 treatment showed slightly increased 4R:3R tau ratio, quantified to be 32 ± 7% higher than control group (Figure 9). However, this change was not statistically significant, and 3 nM OA treatment did not induce a significant shift in the 4R:3R tau ratio either (one-way ANOVA, *p* > 0.05). This indicates that the observed physiological effect of Sphinx31 on outgrowth is not dependent on alternative splicing of the *MAPT* gene. The vehicle for both OA and Sphinx31 is DMSO, thus all values were normalised to 0.02% DMSO treated control group.

## 4. Discussion

There are numerous *in vitro* models for AD that incorporate different aspects of its pathology. To investigate the neuroprotective properties of VEGF-A isoforms, it was necessary to develop a robust neurotoxicity assay that consistently presented concentration-dependent cell death within a reasonable range and that could be rescued by neuroprotective agents.

### 4.1. VEGF-A_165_ isoforms increase viability of SHSY5Y cells in oxidative stress *in vitro* model

Hydrogen peroxide was used to create an oxidative stress model in SHSY5Y cells. In both SHSY5Y and N2a cell lines, NGF treatment evoked a significant increase in cell viability. When treated with either VEGF-A_165_a or VEGF-A_165_b, SHSY5Y cells demonstrated a similar increase in metabolic activity comparable to that without H_2_O_2_. This is in alignment with other studies, which have demonstrated VEGF-A dependent neuroprotection (Beazley-Long et al., 2013) and supports the hypothesis that VEGF-A treatment can increase neuronal viability in the presence of oxidative stress. There was not an observable difference in the effect of alternative splicing isoforms which suggests that despite having opposing effects on angiogenesis (Nowak et al., 2010) both splicing families maintain neuroprotective effects in SHSY5Y cells.

However, VEGF-A induced increase in cell viability was not reproduced in N2a cells. This could be attributed, at least in part, to the smaller cell viability window where relatively low concentrations of hydrogen peroxide (e.g., 50 μM) reduced cell viability to less than half of control. There is limited published data surrounding VEGF-A function in N2a cells, but it has been reported that inhibition of VEGF-A with a neutralising antibody increases N2a cellular viability under hypoxic conditions (Saraswat et al., 2015). This finding was related to maintaining normal levels of VEGF-A since it is upregulated by hypoxia via hypoxia-inducible factor-1 (HIF-1). Since HIF-1 is also known to be upregulated by reactive oxygen species, including H_2_O_2_ (Bonello et al., 2007) it could be postulated that VEGF-A was already upregulated and no longer exerting cytoprotective effects, explaining why we could not measure a significant change in N2a viability. The VEGF-A_165_b isoform did not significantly affect N2a viability either, despite evidence that VEGF-A_165_b can negatively regulate VEGFR2 expression (Ballmer-Hofer et al., 2011) which would arguably counteract HIF-1 induced VEGF-A activity. However, VEGF-A_165_b remains a partial agonist of VEGFR2 (Kawamura et al., 2008) and is dependent on VEGFR2 to elicit neuroprotection (Beazley-Long et al., 2013). Therefore, it is not appropriate to compare VEGF-A_165_b mediated neuroprotection with that related to VEGF-A neutralisation. It is also important to note that Saraswat et al. did not measure VEGFR2 expression or activation, and it is likely that blocking VEGF-A activity would have caused upregulation of VEGFR2 through the clathrin-mediated pathway (Bruns et al., 2010). VEGFR2 trafficking could also explain the lack of response seen in N2a cells if HIF-1 induced VEGF-A expression resulted in VEGFR2 internalisation. For future work, and more comprehensive understanding of neuronal VEGF-A signalling, treatment with recombinant VEGF-A isoforms could be repeated in the presence of an VEGFR2 inhibitor.

### 4.2. Both types of VEGF-A_165_ isoforms increase viability of SHSY5Y cells in amyloid *in vitro* model

The amyloid *in vitro* model was able to induce loss of viability in N2a cells and a significant reduction in SHSY5Y cells. This observation was consistent with previously published work optimising treatment methods of amyloid in various states (Stine et al., 2011). In its unaggregated state, 1 μM amyloid was found to decrease N2a cell survival. In other studies carried out in SHSY5Y cells, amyloid treatment between 1 and 10 μM was successfully used to induce neurotoxicity (Stine et al., 2011; Hettiarachchi et al., 2014; Zheng et al., 2014). As seen in Figure 3, both VEGF-A_165_a and VEGF-A_165_b completely rescued the amyloid-induced decrease in SHSY5Y cell viability. This complements results from the oxidative stress *in vitro* model and confirms that both splicing isoforms are able to rescue neurotoxic effects associated with the accumulation of amyloid.

### 4.3. VEGF-A_165_a increases neurite outgrowth of SHSY5Y cells in tau hyperphosphorylation *in vitro* model

In AD and many other tauopathies, NFTs result from the hyperphosphorylation of tau and its aggregation within neurones (Bancher et al., 1989). Regulation of tau function is dependent on glycogen synthase kinase 3β (GSK-3β) and protein phosphatase 2A (PP2A) enzymes which play a role in tau phosphorylation and dephosphorylation, respectively, (Gong and Iqbal, 2008). In fact, PP2A activity is decreased by ~30% in AD brain (Gong et al., 1995), highlighting its importance in the regulation of normal tau function. Okadaic acid (OA) has been used a potent inhibitor of PP2A to induce tau hyperphosphorylation *in vitro* (Martin et al., 2011). In the development of this neurotoxicity model (as seen in Figure 4), we showed that OA decreases neurite outgrowth of SHSY5Y cells in a concentration-dependent manner. This neurotoxic effect of OA is in alignment with observations from similar studies using OA in SHSY5Y cells (Metin-Armagan et al., 2018; Boban et al., 2019).

Okadaic acid treatment has also been shown to upregulate VEGF-A expression (Wakiya and Shibuya, 1999), which is closely related to increased activity of HIF-1 activated by the mTOR pathway (Kim et al., 2009). While PP2A inhibits ribosomal protein S6 kinase (S6K) activity, VEGFR2 promotes mTOR signalling and PKC activation, which positively regulates S6K. Therefore, OA inhibition of PP2A significantly increases VEGF-A mediated PKC activation and S6K activity (Edelstein et al., 2011) which is yet further augmented by treatment with recombinant VEGF-A. As well as controlling protein synthesis, S6K activity is known to signal cell survival (Harada et al., 2001) which may account (or at least contribute towards) the cytoprotective properties of VEGF-A. In this study, VEGF-A_165_a treatment amplified neurite outgrowth in the absence and presence of OA; this can be observed in Figure 6, which shows an upward shift in SHSY5Y neurite outgrowth in the presence of 0–3 nM OA. This could be mediated by an upregulation of S6K activity, suggesting that VEGF-A could compensate for loss of neuronal function related to tau hyperphosphorylation. When coupled with previous experiments that showed recovery of amyloid-induced decrease in cell viability, it can be concluded that VEGF-A is protective against both forms of AD-related neurotoxicity.

### 4.4. VEGF-A_165_b recovers the decrease in neurite outgrowth of SHSY5Y cells in tau hyper-phosphorylation *in vitro* model

As opposed to VEGF-A_165_a, a full agonist of endothelial VEGFR2 that stabilises its interaction with NP-1, VEGF-A_165_b is a partial agonist of endothelial VEGFR2 that is unable to bind NP-1 and promotes internalisation and degradation of VEGFR2 (Ballmer-Hofer et al., 2011). Consequentially, VEGF-A_165_a binding leads to PKC activation while VEGF-A_165_b binding does not (Kisko et al., 2011; Hulse et al., 2014). Since PKC is a negative regulator of Akt, its inhibition has been shown to increase Akt phosphorylation (Edelstein et al., 2011). Akt is known to inhibit GSK-3β activity (Hermida et al., 2017), which in turn phosphorylates tau (Gong and Iqbal, 2008). If VEGF-A_165_b is able to downregulate neuronal VEGFR2 in a similar way to endothelial VEGFR2, it could be postulated that resulting Akt inhibition of GSK-3β protects against tau hyperphosphorylation and could therefore have knock-on effects on tau function.

In this study, VEGF-A_165_b co-treatment alone did not increase neurite outgrowth of SHSY5Y cells. However, VEGF-A_165_b protected against the decrease in neurite outgrowth seen with 1 and 3 nM OA, as observed by the rightward curve shift in Figure 5 where there is no measured decrease in neurite outgrowth compared to control. This indicates that VEGF-A_165_b is not ubiquitously increasing neurite outgrowth but acting directly against the effect of OA. This could potentially occur via the PI3/Akt pathway: if VEGF-A_165_b causes downregulation of VEGFR2 and attenuates PKC activation, it may lead to increased Akt phosphorylation, greater inhibition of GSK-3β activity, and thus reduced phosphorylation of tau. If sufficient to counter-act OA inhibition of PP2A, this could prevent the hyperphosphorylation of tau and thus protect against downstream effects, including decrease in neurite outgrowth.

### 4.5. SRPK1 inhibitor Sphinx31 significantly decreases VEGF-A_165_a:VEGF-A_165_b ratio in SHSY5Y cells

SRPK1 is related to alternative splicing of VEGF-A through phosphorylation of SRSF1 (Harper and Bates, 2008). SRPK1 activity drives nuclear localisation of SRSF1 and promotes selection of the proximal splice site in exon 8 in epithelial cells, producing-A_165_a (Nowak et al., 2008). SRPK1 inhibition reduces phosphorylation of SRSF1 and consequentially increases selection of the distal splice site in exon 8, producing VEGF-A_165_b (Nowak et al., 2010). This has been established through use of SRPK1 inhibiting compounds SRPN340 and Sphinx31 in a variety of tissues, including the kidney (Amin et al., 2011), retinal epithelium (Gammons et al., 2013; Batson et al., 2017), peripheral neurones (Hulse et al., 2014), prostate cancer (Mavrou and Oltean, 2016), and acute myeloid leukaemia (Tzelepis et al., 2018). Consequently, alternative VEGF-A splicing has been related to a number of pathological conditions, including Denys Drash Syndrome, diabetic retinopathy, age-related wet macular degeneration (AMD), neuropathic pain, and cancer. However, Sphinx31 (or SRPK1 inhibition) had not been previously investigated in SHSY5Y cells, used here as a neuronal model. As shown in Figure 7, SRPK1 inhibition does modulate VEGF-A exon 8 splicing in SHSY5Y cells, making them a viable model to investigate the neuroprotective role of VEGF-A isoforms in AD-related neurotoxicity. Consistent with the effect observed in other tissues, both intracellular and extracellular VEGF-A showed a proportional shift towards the VEGF-A_165_b isoform and significant decrease in VEGF-A_165_a:VEGF-A_165_b ratio. Of interest it also changed the secretion level of the two isoforms, a phenomenon previously shown in neuroblastoma cells (Peiris-Pages et al., 2010). It is possible that SRPK1 inhibition could also change the secretion by changing the RNA sub-compartment of the different RNAs (Hamdollah Zadeh et al., 2015).

### 4.6. SRPK1 inhibition recovers OA-induced decrease in neurite outgrowth and recovery is dependent on endogenous VEGF-A_165_b

Similar to observed effects of recombinant VEGF-A_165_b, Sphinx31 treatment was shown to protect against OA-induced decrease in neurite outgrowth. In the presence of 1 and 3 nM OA, Sphinx31 co-treatment significantly increased outgrowth to a level comparable to vehicle control (see Figure 8). This is a novel finding which suggests SRPK1 inhibition is neuroprotective in the OA model for tau hyperphosphorylation. When SHSY5Y cells were co-treated with Sphinx31 and a VEGF-A_165_b-neutralising antibody (Figure 8), amelioration of neurite outgrowth was significantly reduced. As a result, it can be concluded that the neuroprotective effect of Sphinx31 is dependent on VEGF-A_165_b expression, which increases relative to VEGF-A_165_a with SRPK1 inhibition. However, anti-VEGF-A_165_b-treated SHSY5Y cells still showed significantly higher outgrowth than OA control, demonstrating that the Sphinx31 effect is not completely ablated. This suggests that SRPK1 inhibition could be neuroprotective through other targets, not just via VEGF-A alternative splicing.

**Figure 10:**
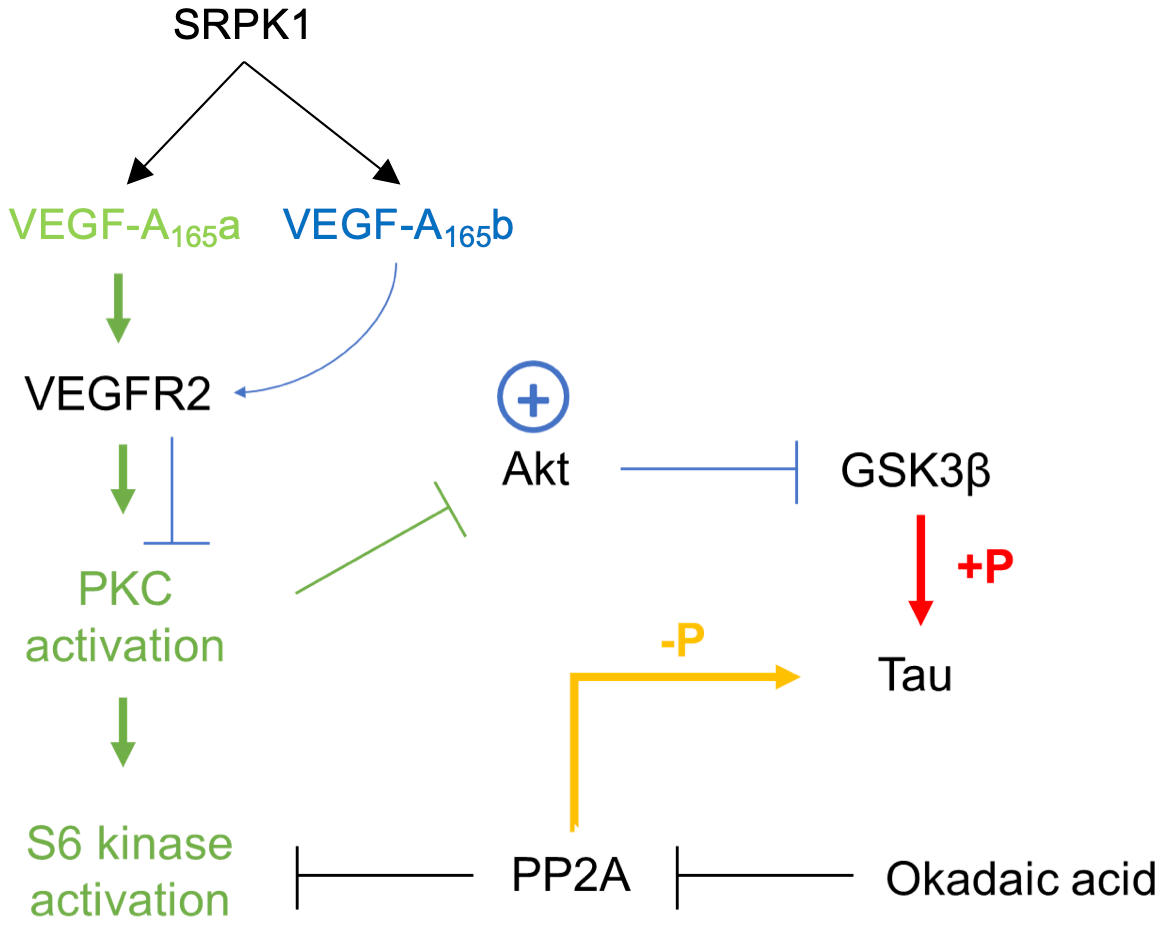
Schematic summarising proposed action of VEGF-A_165_a and VEGF-A_165_b via VEGFR2 binding and full or partial agonism, respectively. Green path represents VEGF-A_165_a activation, blue path represents VEGF-A_165_b activation.

Recombinant VEGF-A_165_b has been reported as a neuroprotective agent in response to numerous insults, including glutamatergic excitotoxicity in hippocampal neurones, chemotherapy-induced cytotoxicity of dorsal root ganglia, and ischaemia-reperfusion injury in retina (Beazley-Long et al., 2013). Therefore, the main findings here are consistent with previous work and confirm that Sphinx31 can be used as a tool to switch VEGF-A splicing and replicate the neuroprotective effect of VEGF-A_165_b in the OA model for tau hyperphosphorylation. Beazley-Long et al. (2013) reported that the neuroprotective action of VEGF-A_165_b in hippocampal neurones was mediated through VEGFR2, as it was blocked by VEGFR2 inhibitors but maintained with selective R1 inhibition. It was also found that hippocampal neuroprotection was unaffected by inhibition of PI3K or p38 MAPK, supporting the previously discussed hypothesis that VEGF-A_165_b acts as a partial agonist at VEGFR2 and does not activate the mTOR pathway (Ballmer-Hofer et al., 2011). Similarly, Sphinx31-mediated VEGF-A_165_b expression seems to act against canonical VEGF-A_165_a signalling, preventing activation of PKC and phosphorylation of PI3K (Harper and Bates, 2008). Therefore, Sphinx31 is likely to exert neuroprotection through another pathway. Beazley-Long et al. (2013) suggest involvement of MEK1/2 pathway because VEGF-A_165_b was shown to induce Erk1/2 phosphorylation, which has been independently shown to ameliorate neuronal damage (Bhowmick et al., 2019). However, activation of Erk1/2 is thought to be altered in AD pathology and has been related to hyperphosphorylation of tau and increased NFT burden (Pei et al., 2002; Veeranna et al., 2004). Considering OA-induced neurotoxicity is based on tau phosphorylation, it would be contradictory to suggest that VEGF-A_165_b-mediated neuroprotection occurred through a mechanism that is known to further phosphorylate tau. It is possible therefore that VEGF-A_165_b (and by extension Sphinx31) dependent neuroprotection occurs through aforementioned inhibition of GSK-3β activity (Figure 10). As this hypothesis is mostly based on evidence from endothelial cells, it would be necessary to empirically test VEGF-A_165_b binding to neuronal VEGFR2 and activation of downstream targets, most notable GSK-3β, in SHSY5Y cells to definitively conclude how neuronal VEGF-A_165_b functions.

There are some limitations to this study. We have only examined one cell line, and additional work on Sphinx31 could focus on investigating its effect on primary neurons such as those used by Beazley-Long et al. (2013), and it is not known whether Sphinx31 is neuroprotective in these contexts. The most closely related data published on the effect of Sphinx31 in nervous tissue identified its use for analgesia: Treatment of primary afferents and resultant shift towards VEGF-A_165_b expression was found to lower VEGF-A_165_a mediated sensitisation of mechanical nociceptors to stimulation (Hulse et al., 2014). Together with data presented here, this identifies SRPK1 inhibition as an interesting therapeutic target in the CNS as well as the PNS, with potential applications in neuroprotection. An additional limitation is that we have measured the protein isoform switch by ELISA using isoform selective antibodies, but it is possible that these ELISAs also detect VEGF-A_121_b or VEGF-A_189_b, and VEGF-A_121_a or VEGF-A_189_a, respectively, so immunoblotting would be useful. However, the VEGF-A_xxx_a antibody (a Fab fragment) is not optimised for immunoblotting and would need to be modified to do so. With regards to AD, it would be extremely useful to extend the findings on VEGF isoform neuroprotection on OA induced effects to other neuronal types, perhaps using neurons differentiated from iPSC cells. It would also be interesting to explore the effect of Sphinx31 on other aspects of its pathology, perhaps using the AD transgenic mouse model, and determine whether *in vitro* results translate *in vivo*. Finally, to show the specificity of the proposed mechanism it would be useful to reinforce the proposed mechanism, for example, by evaluation of the effect of siRNA- or CRISPR-mediated inactivation of SRPK1 and VEGF.

## Data availability statement

The raw data supporting the conclusions of this article will be made available by the authors, without undue reservation.

## Author contributions

LD, DB, RA, and KM contributed to conception and design of the study. TH and JM synthesised the Sphinx31. RA undertook the experiments and wrote the first draft of the manuscript. RA, LD, and DB undertook analysis. DB and LD wrote the sections of the manuscript. All authors contributed to the article and approved the submitted version.

## Funding

This work was funded by the University of Nottingham BBSRC Doctoral Training Programme (Grant BB/M008770/1).

## Conflict of interest

LD, DB, and JM are founders and stock-holders in Exonate Ltd., a company that is developing SRPK1 inhibitors for clinical use. LD and JM are founders and stockholders in Emenda Therapeutics, a company that is developing splicing factor kinase inhibitors for therapeutic use.

The remaining authors declare that the research was conducted in the absence of any commercial or financial relationships that could be construed as a potential conflict of interest.

## Publisher’s note

All claims expressed in this article are solely those of the authors and do not necessarily represent those of their affiliated organizations, or those of the publisher, the editors and the reviewers. Any product that may be evaluated in this article, or claim that may be made by its manufacturer, is not guaranteed or endorsed by the publisher.
